# The Effects of Asymmetrical Guanxi Perception on Job Burnout: Task Conflict, Relationship Conflict, and Process Conflict as Mediators

**DOI:** 10.3389/fpsyg.2021.625725

**Published:** 2021-06-15

**Authors:** Hao-Kai Hung

**Affiliations:** Department of Business Administration, Yango University, Fuzhou, China

**Keywords:** asymmetrical guanxi perception, task conflict, relationship conflict, process conflict, job burnout

## Abstract

In Chinese organizations, individuals benefit in many ways from having good guanxi. For employees, however, guanxi also comes with well-documented negative effects. Until recently, the guanxi investigated in these studies was always of a substantial nature. The negative effects of non-substantial guanxi (in effect, the absence of real guanxi), such as the guanxi of misunderstandings for those who lack good guanxi, have not been examined. This study investigates how the existence of asymmetrical guanxi perception for an employee (i.e., when other people believe that good guanxi exists between a manager and an employee, but the employee disagrees with the belief that he/she has good guanxi with his/her supervisors) contributes to job burnout for that employee due to task conflict, relationship conflict, and process conflict. A cross-sectional data sample was collected from 363 employees of 10 hotels in Taiwan. Structural equation modeling results suggest that relationship conflict is the most powerful mediator affecting the relationship between asymmetrical guanxi perception and job burnout. The results provide insights for researchers interested in the mechanism of how asymmetrical guanxi perception induces employee job burnout while generating useful implications for managers charged with reducing such burnout.

## Introduction

It has been said that guanxi is essential for thriving within a society based on Chinese norms (Pine, 2002; Chen and Chen, 2004; Kivela and Leung, 2005; Hon and Lu, 2016; Miao et al., 2020). Though guanxi has both positive and negative effects, the present study focuses solely on its negative effects. Past studies on the negative effects of guanxi have indicated that they include adverse outcomes, such as procedural injustice perceptions, violation of organizational procedures, negative externalities, cronyism, corruption, job burnout, and an erosion of trust in authority (Gold, 1985; Chu and Ju, 1990; Pye, 1995; Bian, 1997; Leana and Rousseau, 2000; Chen et al., 2004, 2011; Khatri et al., 2006; Gorgievski and Hobfoll, 2008; Chen and Chen, 2009; Hu et al., 2016; Zhang and Gill, 2019; Liu and Jia, 2020; Xu et al., 2020). However, the guanxi investigated in these studies was always substantial; the negative effects of non-substantial guanxi, such as the guanxi of erroneous perceptions, were not investigated.

Guanxi of erroneous perceptions describes the phenomenon in which a peer group regards an individual as being within the inner circle of management, though that individual perceives himself/herself as not actually within that inner circle. In this situation, the individual’s evaluation of the preferences and possession of resources created by such an inner circle becomes an invisible burden; the individual enjoys neither these resources nor favoritism from managers, yet that person’s peers suggest that he or she does. This study defines this phenomenon as asymmetrical guanxi perception.

Misunderstandings can easily cause conflicts (Wall and Callister, 1995); asymmetrical guanxi perceptions are conflicts caused by misunderstandings. According to (Coser, 1956, p. 49), “misunderstanding conflicts are nonrealistic conflicts that are not occasioned by the rival ends of the antagonists, but by the need for tension release of at least one of them.” Based on this definition, such conflicts need to be released. There are many ways to release conflicts. This study aims to understand conflict release methods through three different types of conflicts defined by Jehn (1997) as three distinct groups—task conflict, relationship conflict, and process conflict—and to explain the conflict release caused by asymmetrical guanxi perception.

Moreover, the current study proposes that this type of in-group conflict will further result in the job burnout of employees. Job burnout has always been an important issue in the hospitality industry (Kim, et al., 2007; Dai et al., 2021). Employees with job burnout will have diminished work performance and organizational citizenship behavior, which can in turn eventually lead to behaviors that are costly to their organization (Xu et al., 2020). Therefore, it is important for managers to take steps to avoid employee job burnout.

## Literature Review

### Asymmetrical Guanxi Perception

Guanxi can be defined as a special type of relationship comprised of trust, favor, dependence, and adaptation (Wong, 1998). Employees who work in Chinese organizations must pay particular attention to different types of guanxi existing within such an organization. In addition, guanxi in Chinese organizations can be classified as “in-group” or “out-group”; this describes the tendency of managers to divide subordinates according to guanxi and treat or reward subordinates with different guanxi in a different manner (Tsui and Farh, 1997; Zhang and Gill, 2019). Moreover, actual and potential resources are embedded in the guanxi in-group network possessed by individuals or social units, yet these resources can only be exchanged within a guanxi in-group network (Chen and Chen, 2009; Guan and Frenkel, 2019). Since guanxi can bring so many benefits, people living in a society based on Chinese traditions will care about who exist within the so-called inner circle. Living within a Chinese cultural society in which high power distance and asymmetrical information is prevalent (Zhao et al., 2005), it is easy to characterize a group of people who are misunderstood as having in-group guanxi due to the outside perception of them being close to their boss. In fact, they do not actually possess such in-group guanxi; this phenomenon is defined in our research as “asymmetrical guanxi perception.”

### In-Group Conflict

There are three types of distinct in-group conflict—task conflict, relationship conflict, and process conflict (Jehn, 1997). So-called task conflict refers to the development of different perspectives for work during the discussion among group members or task execution (Jehn, 1995). If group members with different perspectives do not achieve harmony, reach a consistent consensus, or integrate their perspectives, conflicts can arise among them (Jehn and Bendersky, 2003). Relationship conflict is defined as a disharmony of interpersonal relationships which can involve tension, disputes, and even hatred among group members (Jehn, 1995). Jehn and Mannix (2001) define process conflict as disputes regarding how to complete a task. Process conflict is associated with the responsibilities and resource authorization borne by different people.

### Job Burnout

Job burnout is a prolonged response to chronic emotional and interpersonal stressors on the job (Maslach, Schaufeli et al., 2001), a major issue within the service industry (e.g., Gorgievski and Hobfoll, 2008; Jin et al., 2015; Hu et al., 2016; Yang et al., 2019; Liu and Jia, 2020; Xu et al., 2020). The most prevailing belief about job burnout is the definition provided by Maslach and Jackson (1981) who suggested that job burnout is a syndrome with three features: (1) emotional exhaustion, characterized by the over-consumption of individual emotional resources, exhaustion, and loss of energy; (2) depersonalization, a negative and indifferent attitude toward who and what individuals serve; and (3) diminished personal accomplishment as characterized by the tendency to develop a negative evaluation toward self, a sense of helplessness, and decreased self-esteem. However, Demerouti et al. (2001) suggested that the syndrome of job burnout should include two dimensions: exhaustion and disengagement from work. Exhaustion is defined as the outcome of excessive consumption of physical strength, emotion, and perceived tension. Disengagement from work is defined as estrangement from work and development of a negative attitude toward work objectives, work consent, and even the entirety of the work itself, such as the lack of interest in one’s work, the feeling of lack of challenges in one’s work, underestimation of work value, and mechanical execution of work; it is not simply the attitude toward people involved in work (guests). The research subjects in question were employees working in the hospitality industry; consequently, this study adopted the scale developed by Demerouti et al. (2001). As employees in the hospitality industry include the front desk service personnel who directly serve guests, as well as the service personnel cleaning hotel rooms and environment behind the desk, it is more suitable to use a scale including physical strength, emotion, and perception to perform investigations and further explore their relationships within group conflicts.

### The Mediating Role of In-Group Conflict in the Relationship Between Asymmetrical Guanxi Perceptions and Job Burnout

Regarding the effect of in-group conflict caused by asymmetrical guanxi perceptions and in-group conflicts on job burnout, the present study suggests that the relationship can be explained as follows. Firstly, the effect of task conflict on employees who are perceived to be in-group members can be explained by using the self-categorization theory. According to the self-categorization theory (Billig and Tajfel, 1973), peers will treat employees in their outer circle with hostility, viewing them as members of a different group due to the difference in their relationship with their supervisors (Jehn et al., 1999). The work habits of misunderstood employees may be negatively evaluated and criticized by other group members due to such guanxi, which therefore results in task conflict. As such, when a misunderstood employee suggests that he/she has a better way of working, makes a proposal, and attains success, and other peers attribute such success to good guanxi between the employee and the manager, there will be a negative effect on the employee’s perceptions of his or her own abilities which may further cause him or her to experience pressure (Yang and Mossholder, 2004) due to task conflict and eventually experience the exhaustion of job burnout.

In terms of relationship conflicts induced by asymmetrical guanxi perception, when an employee suggests that the guanxi between himself/herself and the manager is different from that between another employee and the manager, relationship conflict rooted in jealousy and even hatred may arise due to the existence of different levels of guanxi. In the collectivist Chinese society, the strategy of avoiding jealousy is usually excessive humility or belittling of the self (Schoeck, 1969, p. 55). Such performances are culturally appropriate behaviors. However, when those who are misunderstood suggest that such guanxi does not actually exist and thus fails to engage in the excessive humility or self-belittling as expected by others, they will be viewed as lacking tact. As a result, they are envied, and relationship conflicts arise. When this happens, his colleagues will use different moral standards to judge the behavior of those who have become jealous; as a result, what was originally right becomes wrong due to differing standards, causing the employees to lose interest in the job and suffer from job burnout as a result. This relationship can be well explained using the moral exclusion theory (Opotow, 2011). Moral exclusion refers to the exclusion of other individuals or groups from one’s own “moral community” (Staub, 1989), and in other words, it means viewing others as lying beyond the boundaries within which moral values and the rules of justice and fairness apply. Based on the moral exclusion theory, people involved in relationship conflict have different attitudes toward opponents involved in the conflict because they are excluded from their “moral community.” They may be unwilling to accept the idea of opponents altogether (Jehn, 1995) thus causing the misunderstood party to lose interest in work and further develop job disengagement (one dimension of job burnout). In addition, exposure to interpersonal conflicts will increase employees’ negative emotional status, which will be further manifested as hostile behaviors (Spector et al., 2006). As a result, existing within such a hostile environment makes one more likely to suffer from exhaustion (another dimension of job burnout).

As for process conflicts caused by asymmetrical guanxi perception, when members of a group suggest that the unequal distribution of job responsibilities is caused by the fact that there is good guanxi between an employee and the manager, the person who is misunderstood will experience the problem of others believing that the distribution of responsibilities is unfair. This type of problem could increase the anger, hostility, and negative attitudes of others (Jordan et al., 2006; Greer and Jehn, 2007), thus creating process conflicts for the misunderstood employee. This result, in turn, causes the employees to accept peers’ attitudes toward unfair evaluation of the parties’ inputs and outputs erroneously believing that many aspects of his resources or work results have been obtained due to guanxi. In the end, this could cause such employees to lose interest in work and subsequently lead to frustration (two dimensions of job burnout). This relationship can be well explained by the effort-reward imbalance model proposed by Siegrist (1996). The main concept of the effort-reward imbalance model includes job effort and job reward, emphasizing the fairness and rationality of efforts and rewards in the social exchange process. The state of imbalance between high job effort and low reward will cause a stress response. Job efforts include external and internal efforts. External efforts refer to individuals’ efforts and responsibilities made in response to the demands of external situations. The internal effort is also defined as excessive involvement in one’s job—namely, the desire to obtain control over one’s job and affirmative responses. Rewards include money, self-esteem, and control of social status. Based on this model, a misunderstanding of guanxi will lead to a state of low reward from one’s colleagues. Such colleagues will attribute all the good performances of an employee to his/her access to more resources and/or the manager’s favoritism, further causing high job effort and low rewards and resulting in the development of nervous responses and diseases which then further lead to a loss of interest in work and frustration.

Based on the discussions above, this study proposes the following hypotheses:

H1: Task conflict has a mediating effect on the relationship between asymmetrical guanxi perception and job burnout.

H2: Relationship conflict has a mediating effect on the relationship between asymmetrical guanxi perception and job burnout.

H3: Process conflict has a mediating effect on the relationship between asymmetrical guanxi perception and job burnout.

## Materials and Methods

### Samples and Procedure

This study collected data from 2,297 employees of 19 departments in 10 hotels located in Taiwan, China. The reason why we choose this industry to investigate this problem is because the hotel industry requires a high degree of cooperation among employees, which is prone to various conflicts and job burnout (Hon et al., 2013). Based on Patton (2015), purposive sampling assists researchers in selecting information-rich cases for study—as such, purposive sampling was used in this project. In consideration of personal privacy, the authors of this study explained the purpose of the research to the human resource managers of each hotel in order to ensure the confidentiality of their answers. Human resource managers were asked to select employees from different departments who might be in-group members of department heads. Under the assistance of human resource managers in each hotel, this study obtained a list totaling 450 employees who met the inclusion criteria (employees who might be misunderstood as having a good relationship with their managers—namely, employees perceiving a lack of good Guanxi) and a total of 1,847 colleagues. The data presented here were collected over two time periods. The first sample was used to identify subjects who met the inclusion criteria and evaluated their in-group guanxi with supervisors, conflict with colleagues, and job burnout. The second set of data was collected to attain department colleagues’ evaluations of in-group guanxi between the subjects and their department managers. This study distributed questionnaires to employees meeting the inclusion criteria and colleagues in their departments according to this list. The author of this study compiled the questionnaire before distribution, and the human resource managers assisted in matching the questionnaires where department colleagues evaluated the research subjects. The author and assistants investigated the subjects who met the inclusion criteria and their colleagues during slack seasons in hotels from 2015 to 2016. The subjects who met the inclusion criteria were first asked whether they had been misunderstood as having good guanxi with supervisors and thus experienced problems. If the answer was “Yes,” they were asked to respond to questions regarding: the guanxi in-group perceived by them; job conflict; relationship conflict; process conflict; and job burnout. Next, department colleagues were asked to evaluate the in-group guanxi between the subjects meeting the inclusion criteria and department managers. A total of 363 subjects meeting the inclusion criteria completed and returned the questionnaires on site, with a return rate of 80.67%. Colleagues returned 1,150 questionnaires, at a return rate of 56%. This study excluded unmatched questionnaires, then conducted a questionnaire survey. A total of 1,110 matched questionnaires completed by the research subjects meeting the inclusion criteria and their colleagues were obtained. This study offered a coupon for coffee as a reward for each participant. In terms of gender, the majority of respondents were female (59%). In terms of age, the largest percentage was 21–25 years old (35%), followed by the group aged 26–30 years old (25%). In terms of educational background, most respondents had a bachelor’s degree (67%), followed by those having an associate degree (23%). The mean seniority was 4.6 years.

### Measures

All the data were presented in Chinese. Therefore, this study used the translation and back-translation procedures recommended by Brislin (1980).

#### Asymmetrical Guanxi Perception

This study used the six items developed by Law et al. (2000) to evaluate asymmetrical guanxi perception. An example item is “My supervisor invites me to his/her home for lunch or dinner.” The employees answered questions about their guanxi with department managers, and their colleagues evaluated the guanxi between the subjects meeting the inclusion criteria (the misunderstood employees), respectively. The score of asymmetrical guanxi perception of each misunderstood employee was obtained as follows: [(score evaluated by the employee − score evaluated by the colleagues)/number of matching pairs]. This study used a 7-point scale (1 = strongly disagree and 7 = strongly agree) for evaluation, where Cronbach’s *α* = 0.86.

#### Task Conflict

The employees receiving the interviews completed the scale (three items) developed by Jehn (1995), in which a 5-point scale was used for evaluation (1 = Strongly disagree and 5 = Strongly agree) and Cronbach’s *α* = 0.82. A typical item reads “How frequently are there conflicts about ideas in your work unit?”

#### Relationship Conflict

The employees receiving the interviews completed the scale (three items) developed by Jehn (1995) with a 5-point scale used for evaluation (1 = strongly disagree and 5 = strongly agree) and Cronbach’s *α* = 0.81. An example item reads “How much emotional conflict is there among members in your work unit?”

#### Process Conflict

The employees undergoing interviews completed the scale (three items) developed by Shah and Jehn (1993), where Cronbach’s *α* = 0.86. One such item reads “To what extent did you disagree about the way to do things in your work group?”

#### Job Burnout

The burnout scale (Demerouti et al., 2001) refers to the two core facets of emotional exhaustion and disengagement (or cynicism). We used four items for each facet, with answers ranging from 1 (strongly disagree) to 5 (strongly agree). The exhaustion scale internal consistency was Cronbach’s *α* = 0.90. The disengagement scale internal consistency was Cronbach’s *α* = 0.86. An example item for the exhaustion scale was “job demand to physical workload.” An example item for the disengagement scale was “job resource to feedback.”

#### Control Variables

We asked for the gender, education, and length of work tenure of employees in the questionnaire.

## Tests of Hypotheses

### Reliability and Validity Assessment

Means, standard deviations, correlation coefficients, and reliability estimates of all variables are shown in Table 1. The reliability of all scales is satisfactory, with Cronbach’s *α* scores ranging from 0.81 to 0.90.

**Table 1:** Means, standard deviations, and correlations.

S. No.	Variable	M	SD	1	2	3	4	5	6	7	8	9
1	Gender^b^	0.46	0.50	–								
2	Education^c^	3.85	0.54	0.00	–							
3	Tenure^d^	4.6	1.70	−0.13	−0.14^*^							
4	Asymmetrical guanxi perception	3.89	0.58	0.09	−0.04	0.08	0.86^a^					
5	Task conflict	3.86	0.65	−0.07	−0.02	0.10	0.70^*^	0.82				
6	Relationship conflict	3.88	0.67	−0.10	0.06	0.03	0.74^*^	0.60^*^	0.81			
7	Process conflict	3.60	0.69	−0.02	−0.07	0.05	0.52^*^	0.53^*^	0.41^*^	0.86		
8	Job burnout—exhaustion	3.89	0.64	−0.11	−0.03	0.02	0.75^*^	0.69^*^	0.79^*^	0.49^*^	0.90	
9	Job burnout—disengagement	3.86	0.64	−0.09	−0.04	0.04	0.72^*^	0.66^*^	0.69^*^	0.53^*^	0.81^*^	0.86

* *p* < 0.01 (two tailed).

a Scale reliabilities reported on the diagonal. *N* = 363.

b female = 0, male = 1.

c Under high school = 1, high school = 2, college = 3, under graduate = 4, and graduate = 5.

d Organizational tenure was measured in years.

### Measurement Model

A measurement model of all multi-item measures was subjected to confirmatory factor analysis (CFA) in order to assess the convergent and discriminant validity of all constructs. In this model, each item was loaded on its appropriate factor, and we included job burnout as a second-order latent factor predicting its two dimensions of exhaustion and disengagement. We compared the fit of four different models: a one-factor model; a five-factor model; a six-factor model; and the hypothesized seven-factor model (asymmetrical guanxi perception, task conflict, relationship conflict, process conflict, job burnout-emotional exhaustion, job burnout-disengagement, and overall job burnout). The seven-factor model fit was acceptable *χ*^2^(218) = 710.68; CFI = 0.91; TLI = 0.90; RMSEA = 0.08; and SRMR = 0.05 (Hu and Bentler, 1999). In this model, all factor loadings were significant (*p* < 0.05), all standardized factor loadings were larger than 0.60, and correlation coefficients among all latent factors were substantially smaller than 1.0. The seven-factor model fit was significantly better than that for the six-factor model (*χ*^2^ (222) = 867.22, *p* < 0.01; CFI = 0.89, TLI = 0.87; RMSEA = 0.09, SRMR = 0.05), the five-factor model (*χ*^2^ (225) = 1157.62, *p* < 0.01; CFI = 0.84, TLI = 0.82; RMSEA = 0.11, SRMR = 0.06), or the one-factor model (*χ*^2^ (230) = 1327.65, *p* < 0.01; CFI = 0.81, TLI = 0.79; RMSEA = 0.12, SRMR = 0.07), supporting our measurement model.

### Structural Model

**Figure 1:**
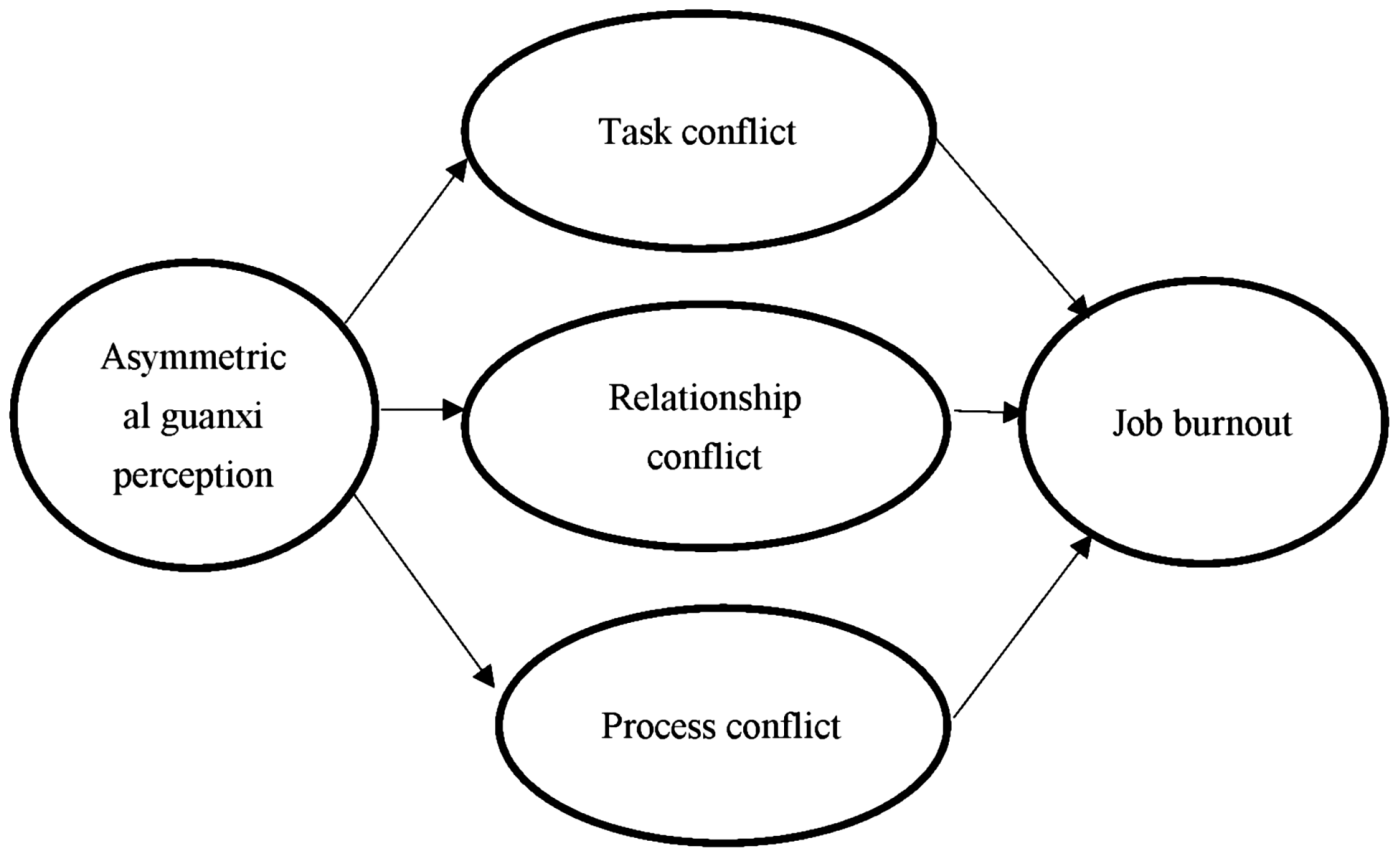
Hypothesized model.

Our purpose was to explore the mediating mechanisms by which asymmetrical guanxi perception leads to job burnout. To examine multiple mediation effects simultaneously, we used Lau and Cheung’s (2012) procedure to assess and compare specific indirect effects in complex latent variable models. All variables in Figure 1 were considered to be first-order latent variables, except for job burnout, which was a second-order latent variable of the two first-order dimensions of exhaustion and disengagement. The analyses were conducted using Mplus 7.4 with maximum likelihood estimation.

**Figure 2:**
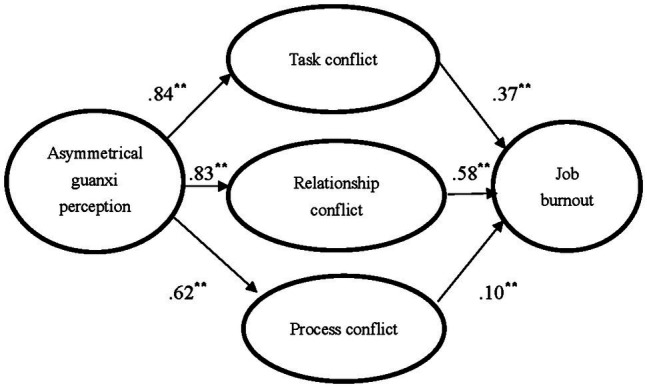
Structural equation modeling results for hypothesized model. ^*^p < 0.05, two-tailed; ^**^p < 0.01, two-tailed.

Overall, the fit statistics for our theoretical model indicate a good fit: *χ*^2^ (288) = 875.404, *p* < 0.01; CFI = 0.90; TLI = 0.89; RMSEA = 0.08. We present the visual representations of our results regarding the hypothesized model in Figure 2. Regarding the relationships between the independent variable and the mediators, asymmetrical guanxi perception is significantly related to all three mediators: task conflict (*B* = 0.84, *p* < 0.05); relationship conflict (*B* = 0.83, *p* < 0.05); and process conflict (*B* = 0.62, *p* < 0.05). For the relationships between each mediator and the dependent variable, all are significantly related to job burnout (task conflict → job burnout, *B* = 0.37, *p* < 0.05; relationship conflict → job burnout, *B* = 0.58, *p* < 0.05; process conflict → job burnout, *B* = 0.10, *p* < 0.05). The influence of control variables on job burnout is significant only with regard to gender (*B* = −0.08, *p* < 0.05).

Significance tests of each indirect effect were accomplished *via* bootstrapping procedures that created a 95% of the confidence interval around the indirect effect estimates. The results show that all of the indirect effects are significant (refer to Table 2). Thus, we conclude that task conflict, relationship conflict, and process conflict fully mediate the relationship between asymmetrical guanxi perception and job burnout. Therefore, hypotheses H1, H2, and H3 are all supported.

**Table 2:** Indirect effects and bootstrapping results.

	Asymmetrical guanxi perception^*^		
	Indirect effect	95% bootstrapped confidence interval	
		Lower	Upper
Task conflict	0.31	0.16	0.44
Relationship conflict	0.46	0.34	0.63
Process conflict	0.06	0.00	0.11

* Based on 2,000 bootstrap samples.

Furthermore, because our proposed model is a multiple mediation model, and we are concerned with the comparison among the indirect effects in the model, we used AGP_TC_JB to represent the indirect effect. We used (AGP_TC_JB
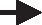
AGP_RC_JB) to represent the difference between two indirect effects. The point estimates of the effect sizes, as well as bootstrap estimates of their confidence intervals based on 2,000 bootstrapping samples, are shown in Table 3. The results of contrasting indirect effects show that (AGP_TC_JB
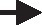
AGP_PC_JB) and (AGP_RC_JB
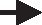
AGP_PC_JB) are statistically significant. A positive (AGP_TC_JB
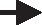
AGP_PC_JB) estimate means that the indirect effects of AGP_TC_JB are larger than the indirect effects of AGP_PC_JB. Similarly, a positive (AGP_RC_JB
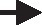
AGP_PC_JB) value means that the indirect effects of AGP_RC_JB are larger than the indirect effects of AGP_PC_JB.

**Table 3:** Contrasting the indirect effects.

	Estimate	95% bootstrapped confidence interval	
		Lower	Upper
^*^AGP_TC_JB 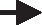 AGP_RC_JB	−0.186	−0.50	0.069
^*^AGP_TC_JB 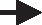 AGP_PC_JB	0.290	0.100	0.480
^*^AGP_RC_JB 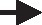 AGP_PC_JB	0.480	0.289	0.675

* AGP, asymmetrical guanxi perception; TC, task conflict; RC, relationship conflict; PC, process conflict; JB, job burnout.

## Discussion

This study investigated the effects of asymmetrical guanxi perception on employee job burnout. A distinguishing feature of the present study is its focus on the mechanism of guanxi on job burnout. The empirical results showed that, overall, the non-substantial effects caused by asymmetrical guanxi perception on employee job burnout are significant and differ from the negative effects of substantial guanxi; this complements the gap left by the research on the substantial guanxi that affects the job burnout (e.g., Gorgievski and Hobfoll, 2008; Hu et al., 2016; Yang et al., 2019; Liu and Jia, 2020; Xu et al., 2020). In fact, the asymmetrical guanxi perception can be seen as negative effects caused by information asymmetry. As a result, to flourish in Chinese society, employees have to pay attention to non-substantial guanxi and take into account the guanxi caused by information asymmetry. However, like Pandora’s box, asymmetrical guanxi perception caused by information asymmetry can create disasters as well as hope; as indicated in past studies, the proper use of asymmetrical guanxi perception caused by information asymmetry can also create benefits (Uzzi, 1999). Future studies may further investigate its positive effects on employees.

This study tested three mediators. These three mediating mechanisms—in-group task conflict, relationship conflict, and process conflict—caused by asymmetrical guanxi perception contributed to the job burnout of misunderstood employees. The results showed that the mediating effect of relationship conflict on asymmetrical guanxi perception and job burnout is most significant because it conforms to the interpersonal harmony valued in Chinese society and the severe outcomes caused by loss of harmony (Leung et al., 2002). The past studies on Chinese society have found that good guanxi with colleagues can be a buffering factor on job burnout (Lee and Akhtar, 2007; Wu and Hu, 2009). By contrast, the lack of good guanxi with colleagues will cause job burnout, as shown in this study. Workplace suffering caused by the pursuit of harmonious relationships in Chinese culture may only exist in Chinese workplace culture. As indicated by Narayanan et al. (1999), the work pressure defined in a particular culture may not exist in another culture. Future studies may further explore the development of interpersonal sources of pressure and the psychological mechanism from the perspective of harmonious ideology or further analyze differences between Chinese and Western organizational contexts.

This study suggests that the significant mediating effect of task conflict represents the fact that the asymmetrical guanxi perception indeed causes significant work problems for misunderstood employees, whose job performance—to their frustration—may be easily attributed to guanxi. These employees tend to experience two frustrations; it is their duty to perform well and their fault for not performing well. For example, when a misunderstood employee achieves success using a better way of working, other people may attribute such success to guanxi instead of to his or her ability. As a result, the misunderstood employee will experience the frustration of self-evaluation, as others believe it is his/her duty to perform well (Swann et al., 2004). Moreover, when other people think that a misunderstood employee could obtain more resources using good guanxi (Chen and Chen, 2009), but he or she does not perform well on the job, he/she will experience the frustration of facing criticism for failing to perform well. Future studies may further explore the effect of this “Catch-22” on different dimensions of employees in work.

This study suggests that the mediating effect of process conflict may be weaker because the unfair distribution caused by guanxi has become a common phenomenon in Chinese society. Leung and Bond (1984) mentioned that this value exists in Chinese society. In addition, Chiu (1989) suggested that fairness in Chinese society merely describes the benefits enjoyed by in-group members, while out-group members are treated differently. Therefore, employees can better adapt to process conflict caused by unfair distribution, and the effect of job burnout caused by this phenomenon becomes less significant.

### Practical Implications

As indicated in this study, job burnout arising from asymmetrical guanxi perception is mainly reflected by task conflict and relationship conflict. However, the conflicts are mainly caused by misunderstandings in connection with information asymmetry. Thus, it is required to date back the asymmetrical perception. To solve problems brought by information asymmetry and the problem of asymmetrical perception, the best and quickest way for employees is to promote relation building and understanding with each other through interactions during office and off-duty hours. Long-term off-duty interaction is an important approach to developing guanxi in Chinese culture, in addition to the interaction during office hours (Chen and Peng, 2008). The best practice to solve the problem of horizontal relations arising from vertical relations is to enhance trust and understanding based on the long-term cultivation of horizontal relations. If the misunderstanding is left alone, the misunderstood person will not only fail to get support from colleagues (Halbesleben, 2006) but also suffer from job burnout caused by task conflict and relationship conflict as described in this study. It is suggested not to clarify the relation through the superior, because the in-group relation between superiors and subordinates is hidden guanxi in the organizational culture of China. The situation will be worse if the supervisor is involved, and others may guess the reason why the supervisor is helping to be simply due to having a good relationship with one’s supervisor.

### Limitations and Directions for Future Research

In terms of research limitations, Luo (2011) developed the scale for in-group guanxi from the perspective of social network analysis, with better measures for the measurement of guanxi. This study did not use this method of measurement because complete network locations could not be obtained without a more complete set of organizational members participating in the measurement of the social network. Therefore, this study used a non-social network analysis scale developed by Law et al. (2000) based on the belief that data collection from departments in different hotels could obtain more extensive results. In addition, this study collected data during a single period. In addition, because the subjects of this study were Chinese people, all forms/scales should better be modified by the Chinese population’s personality. It is suggested that the revised scale tested in China should be used in future research to better conform to Chinese culture. A review of longitudinal data will help better clarify the mechanism behind how job burnout develops; future studies could use the latent growth model to analyze this mechanism. Lastly, this study did not include the effects of control variables. However, given the significance of the results, the stability of the overall trend of these results is likely to be maintained but should be interpreted with caution.

## Conclusion

Our study finds that employees’ asymmetrical guanxi perception will negatively affect employees’ job burnout through task conflict, relationship conflict, and process conflict. Among these three conflicts, the mediation effect of relationship conflict is the most significant. This finding highlights that, in addition to the negative effect of substantial guanxi, non-substantial asymmetrical guanxi perception is another problem that needs to be solved, as it will greatly increase employees’ job burnout through relationship conflicts. Additionally, relationship conflicts in a guanxi-based society, such as China are quite serious issues, so any cause of such conflicts should be taken seriously. We encourage future research examining the positive and negative effects of asymmetrical guanxi perception on employees.

## Data Availability Statement

The raw data supporting the conclusions of this article will be made available by the author, without undue reservation.

## Ethics Statement

The studies involving human participants were reviewed and approved by the University of Taipei. The patients/participants provided their written informed consent to participate in this study.

## Author Contributions

H-KH designed research, performed research, analyzed data, and wrote the paper.

### Conflict of Interest

The author declares that the research was conducted in the absence of any commercial or financial relationships that could be construed as a potential conflict of interest.
